# Comparison of the Effect of Mask Preconditioning With and Without Video Demonstration on Child Induction Behaviour During Inhalational General Anaesthesia: A Randomised Controlled Trial

**DOI:** 10.7759/cureus.94702

**Published:** 2025-10-16

**Authors:** Alexander Jacob, John George Aiyankovil, D Girijanandan Menon, Sharon B Thomas, Varghese Jacob

**Affiliations:** 1 Department of Critical Care Medicine, Malankara Orthodox Syrian Church Medical College, Ernakulam, IND; 2 Department of Critical Care Medicine, Believers Church Medical College Hospital, Thiruvalla, IND; 3 Department of Anaesthesiology, Malankara Orthodox Syrian Church Medical College, Ernakulam, IND; 4 Department of Endocrinology, Malankara Orthodox Syrian Church Medical College, Ernakulam, IND; 5 Department of Anaesthesiology, Government Medical College, Pudukkottai, Pudukkottai, IND

**Keywords:** anaesthesia induction, child behaviour, elective surgery, facemask acceptance, general anaesthesia, inhalational induction, paediatric anaesthesia, parental involvement, preoperative anxiety, video demonstration

## Abstract

Background: Inhalational induction of anaesthesia in children is often complicated by poor facemask acceptance and perioperative anxiety, leading to crying, agitation and distress, which can create negative experiences for both children and parents. Such difficulties have been associated with prolonged induction times, increased anaesthetic risk and adverse postoperative behavioural outcomes. Several non-pharmacological strategies, including flavoured masks, audiovisual distraction and parental presence, have been explored to improve cooperation, with varying success. Mask preconditioning has been proposed as a simple, practical approach to reduce fear; however, the role of structured video demonstration combined with parental and child participation remains underexplored. This study aimed to compare facemask acceptance during inhalational induction of anaesthesia in children aged 4-10 years, with and without video demonstration of mask preconditioning.

Methods: This single-blind, randomised controlled trial was conducted from March to May 2021 at Malankara Orthodox Syrian Church Medical College, Kerala, India. Seventy-five children were recruited; 66 were randomised (35 video and 31 no video). The intervention group viewed a prerecorded two-minute educational video demonstrating mask preconditioning, followed by parental and child simulation. The control group received only a live demonstration. Outcomes included facemask acceptance (assessed using the Child Induction Behavioural Assessment Tool (CIBAT)), time to loss of eyelash reflex (T₁) and time to intravenous (IV) cannulation (T₂). The study was approved by the Institutional Ethics Committee of Malankara Orthodox Syrian Church Medical College, Kolenchery, Kerala, India (approval number: MOSC/IEC/412/2020, date: 17-01-2020) and registered with the Clinical Trials Registry-India (CTRI/2021/03/032389).

Results: Mask acceptance showed no significant difference between groups (p = 0.16). The mean time to loss of eyelash reflex was significantly longer in the video group (237.1 ± 34.4 seconds) compared to the no-video group (217.3 ± 36.9 seconds, p = 0.027). No difference was observed in the time to intravenous cannulation.

Conclusion: Video-assisted mask preconditioning with parental and child simulation did not significantly improve overall facemask acceptance. It was, however, associated with smoother inductions and longer times to loss of eyelash reflex. These findings are preliminary and should be interpreted cautiously in light of the study’s limitations, including reduced sample size and single-centre design. Larger, multicentre studies are required to confirm these results before any consideration of routine clinical application.

## Introduction

General anaesthesia induction in children remains a challenging aspect of perioperative care, particularly in anxious or uncooperative patients [1,2]. Inhalational induction is the most frequently used method due to ease of administration, avoidance of intravenous (IV) cannulation and its general acceptance among children. However, poor mask acceptance is a common barrier that can trigger crying, distress and agitation, ultimately leading to negative perioperative experiences [3,4]. Such difficulties may contribute to increased anxiety for both child and parent, prolongation of induction and greater risk of adverse events. In contrast, a cooperative induction facilitates smooth anaesthesia maintenance, rapid recovery and early discharge, while also reducing stress for the anaesthesia team and caregivers [5,6].

To address these challenges, a variety of non-pharmacological interventions have been developed with the aim of improving mask acceptance and reducing perioperative anxiety. Flavoured anaesthetic masks, designed to make the experience more pleasant, have been used successfully in paediatric practice [5]. Similarly, specially designed child-friendly masks incorporating playful or cartoon-like features have been trialled, with the intention of making the induction process less intimidating [6]. Audiovisual distraction techniques have also shown promise: Berghmans et al. demonstrated that audiovisual aids introduced before induction could reduce parental anxiety [7], while Aydin et al. reported that playful mask use as a premedication strategy improved acceptance [8]. Animated cartoon distractions have further been shown to alleviate perioperative anxiety in children undergoing induction [9].

Beyond distraction techniques, specific strategies are often required for children who are particularly anxious or uncooperative. Tailored behavioural approaches have been suggested to manage such patients, with emphasis on structured preparation and interaction [10,11]. Various inhalational induction methods have also been described, such as the “steal induction,” the incremental tidal volume approach and the single-breath vital capacity technique [12]. Parent-led interventions have been tested as well, with Walker et al. [13] demonstrating that parental involvement in mask exposure before surgery enhanced cooperation and significantly reduced preoperative anxiety. Nonetheless, evidence also indicates that parental presence alone may not suffice in highly anxious children, and additional strategies are often required [11,12].

Standardised assessment tools have further improved the ability to objectively evaluate child behaviour during induction. The Child Induction Behavioural Assessment Tool (CIBAT) provides a validated framework for categorising responses to facemask application and has been increasingly utilised in clinical research to ensure consistency and reproducibility [14]. Such tools allow for better comparison across studies and provide reliable measures of intervention efficacy.

In recent years, audiovisual and educational interventions have received growing attention as preparation methods. Kain et al. showed that family-centred video-based preparation improved perioperative outcomes in children by reducing anxiety and enhancing compliance [15]. Patel et al. reported that distraction with hand-held video games significantly reduced preoperative anxiety in the paediatric population [16]. These findings highlight the potential of multimedia-based strategies to improve acceptance and cooperation during anaesthetic induction.

Despite these advances, there is limited evidence evaluating structured video-based mask preconditioning interventions that integrate video demonstration, parental simulation and child participation. The current study aims to address this gap by examining the effectiveness of a short educational video, combined with parental and child involvement, on improving facemask acceptance during inhalational induction. Specifically, this study compares induction behaviour and cooperation between children exposed to video-assisted mask preconditioning and those receiving standard preoperative preparation, using validated behavioural assessment tools. By using validated behavioural scoring methods, this study seeks to provide evidence for a practical, reproducible and child-friendly strategy that can be easily implemented in routine paediatric anaesthetic practice.

## Materials and methods

Study design

This was a single-blind randomised controlled trial conducted from March to May 2021 at Malankara Orthodox Syrian Church Medical College, Kolenchery, Kerala, India.

Sample size calculation

The sample size was calculated for the comparison of two proportions. Assuming a baseline rate of poor mask acceptance of 50% in the control group, based on previously published studies reporting approximately 21%-50% of children exhibiting moderate to poor mask acceptance during inhalational induction of anaesthesia [17], and expecting a clinically meaningful absolute reduction to 25% in the intervention group (difference of 25 percentage points), the required sample size was calculated. Using a two-sided α = 0.05 and power = 80% (β = 0.20), the estimated sample size was approximately 58 participants per group (calculated using the standard formula for two independent proportions), rounded up to 60 per group (total 120) to allow for dropouts. This yields n ≈ 57.6 per group.

Blinding

The study was described as single-blind. The anaesthesiologists responsible for induction and the independent outcome assessors were blinded to group allocation. Parents and children were aware of their intervention (video or no video), but the anaesthesia team and data analysts remained blinded.

Ethics

The trial was registered with the Clinical Trials Registry-India (CTRI/2021/03/032389) [18]. This study was approved by the Institutional Ethics Committee of Malankara Orthodox Syrian Church Medical College, Kolenchery, Kerala, India (approval number: MOSC/IEC/412/2020, approval date: 17-01-2020; ECR/728/Inst/K/2015/RR-18). The study was conducted in accordance with the ethical standards of the committee and with the 1964 Helsinki Declaration and its later amendments or comparable ethical standards [19]. Written informed consent was obtained from the parents/guardians of all participants before enrolment.

Participants

Children aged 4-10 years, classified as American Society of Anesthesiologists (ASA) physical status I and II and scheduled for elective surgery under general anaesthesia, were eligible. Exclusion criteria included the presence of a pre-existing intravenous cannula, anticipated difficult airway, history of previous surgery or recent respiratory infection.

Intervention

Children in the intervention group viewed a two-minute prerecorded video demonstrating mask preconditioning. The video depicted a child calmly accepting the mask, breathing through it and being reassured by a parent. After the video, parents and children practiced mask simulation using the anaesthesia mask and breathing circuit under the investigator’s supervision. The parent first demonstrated the procedure by gently applying a standard paediatric anaesthesia facemask attached to a breathing circuit (without anaesthetic gases), while reassuring the child and demonstrating calm, normal breathing through the mask. The inflation and deflation of the reservoir bag during each breath were shown to the child to familiarise them with the circuit’s function. The child then repeated the same steps to become comfortable and confident with the process.

The video content was developed by two consultant paediatric anaesthesiologists in consultation with a psychologist and reviewed for accuracy and appropriateness before use. Similar audiovisual interventions have previously been shown to reduce perioperative anxiety in children [7,15,16]. To ensure standardisation, the same video was shown to all participants in the intervention group using the same device and under identical conditions.

Children in the control group received only a live demonstration by the investigator without video. The investigator showed the parent and child how the standard paediatric anaesthesia facemask, attached to a breathing circuit (without anaesthetic gases), is applied over the nose and mouth. The child was encouraged to observe the inflation and deflation of the reservoir bag during normal breathing to become familiar with the equipment.

Randomisation

Participants were randomised using block randomisation. Allocation was concealed in sealed opaque envelopes.

Anaesthesia protocol

On the morning of surgery, participant children were premedicated with oral triclofos sodium (Pedicloryl) 75 mg/kg, administered one and a half hours before transferring the child to the reception area of the operating room. Triclofos was chosen because it is a safe, palatable and commonly used paediatric sedative in India, known for its short sleep onset and minimal adverse effects, as previously reported [20]. The participant child was received in the reception area by a nurse or the anaesthetist involved and an assistant holding the facemask and circuit, as is the standard practice in the institution. The child was either carried or allowed to walk, or transferred on a trolley, depending on his/her wish. Inhalational induction was performed with sevoflurane using the incremental tidal volume technique via facemask, consistent with prior studies [12,13]. All inductions were conducted by the senior author, an experienced consultant anaesthesiologist, to maintain uniformity.

Anaesthetic induction was carried out using the incremental tidal volume method. The procedure began with the application of a paediatric anatomical facemask (T₀) attached to a paediatric breathing circuit delivering 100% oxygen at a flow rate of 6 L per minute. The sevoflurane concentration was increased incrementally by 1% every two to three breaths, up to a maximum of 4%, while the child breathed spontaneously.

The time to mask induction (T₁) was defined as the interval from application of the facemask (T₀) to the loss of eyelash reflex, measured using a stopwatch. The endpoint of anaesthetic induction was defined by loss of eyelash reflex, regular respiration, loss of muscle tone and absence of movement to the trapezius muscle squeeze.

Intravenous (IV) cannulation was performed only after achieving this adequate depth of anaesthesia. The time to IV cannulation (T₂) was defined as the interval from the loss of eyelash reflex (T₁) to successful establishment of intravenous access. IV insertion was never attempted before mask acceptance or under a light plane of anaesthesia.

Outcomes

The primary outcome was facemask acceptance, assessed using the Child Induction Behavioural Assessment Tool (Table 1) [14]. The CIBAT is a validated framework for categorising behavioural responses to facemask application, providing a structured and reproducible assessment during inhalational induction. Behaviour was classified into three categories: smooth (calm and cooperative), moderate (crying or verbal refusal) and difficult (active resistance such as screaming or pushing away). Categories are summarised in Table 1 [14].

**Table 1:** Categories of induction behaviour using the CIBAT Categories of behavioural response during inhalational induction, adapted from Winterberg et al. (2018) (Child Induction Behavioural Assessment Tool) [14] CIBAT: Child Induction Behavioural Assessment Tool

Category	Child behaviour
Smooth	Calm, cooperative, does not resist induction
Moderate	Cries, pulls away, verbal refusal
Difficult	Struggles, pushes mask away, kicks/flails, rigid/limp

Secondary outcomes included time to loss of eyelash reflex (T₁), measured from mask application to disappearance of the reflex, and time to successful intravenous cannulation (T₂), measured from time of loss of eyelash reflex to establishment of intravenous access.

Statistical analysis

Categorical variables (mask acceptance, age distribution and gender distribution) were analysed using the Chi-square test, and results are reported with χ² statistics, degrees of freedom, p-values and effect sizes (Cramer’s V, where appropriate). Continuous variables (time to loss of eyelash reflex and time to intravenous cannulation) were analysed using the unpaired t-test, with results presented as mean ± standard deviation (SD), t statistics, 95% confidence intervals (CIs) and p-values. Statistical analysis was performed using Microsoft Excel (Microsoft Corp., Redmond, WA). A p-value of <0.05 was considered statistically significant.

## Results

**Figure 1:**
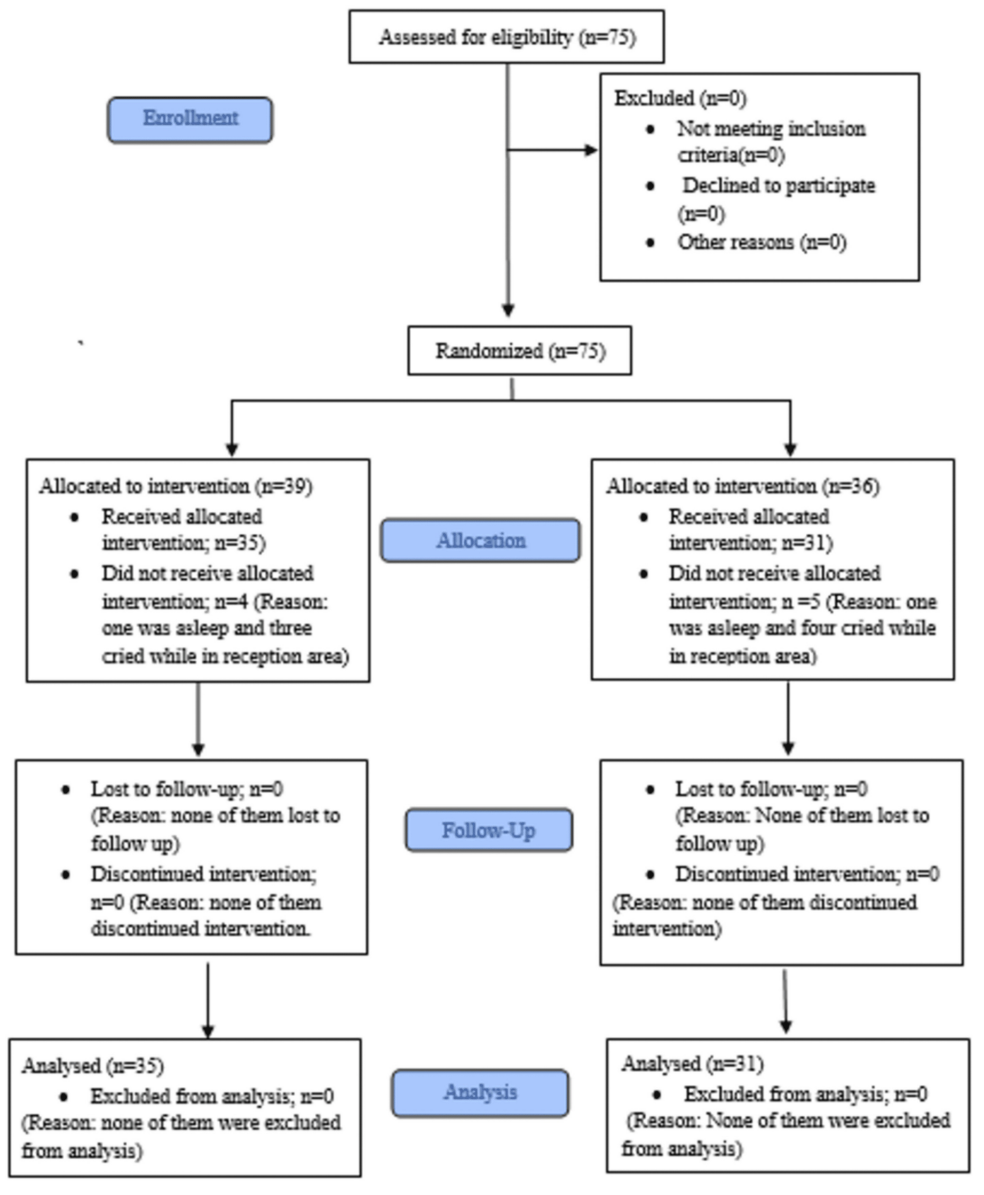
CONSORT flow diagram of participant enrolment, allocation and analysis CONSORT: Consolidated Standards of Reporting Trials

A total of 75 children were enrolled in the study. Nine participants were excluded (six did not meet the inclusion criteria and three declined to participate). Sixty-six children were randomised and completed the study (35 in the video group and 31 in the no-video group). The study flow is presented in Figure 1.

**Figure 2:**
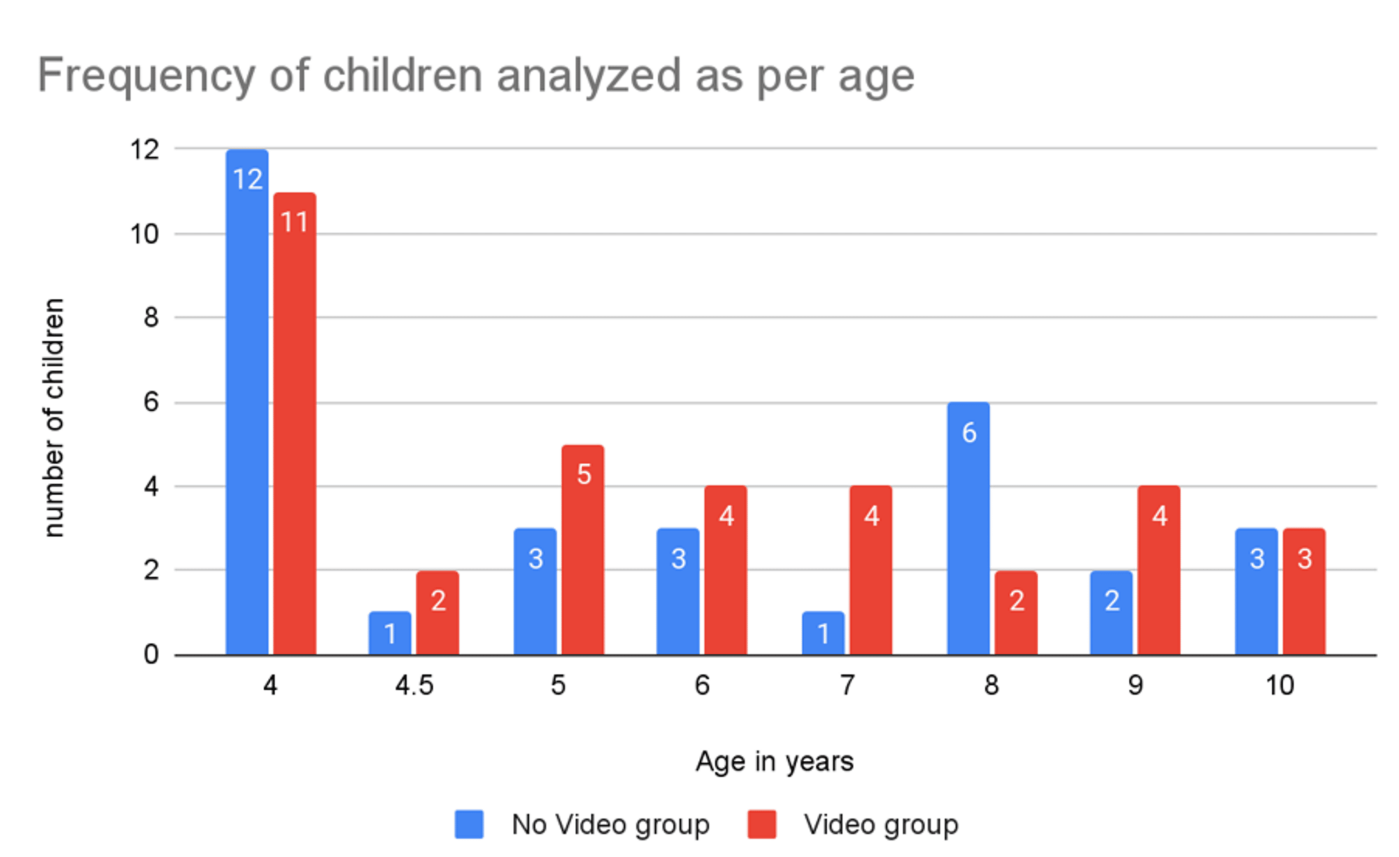
Age distribution of the participants in the video and no-video groups

The age distribution of the participants is summarised in Table 2 and illustrated in Figure 2. Children ranged from 4 to 10 years, with the majority (62.1%) between 4 and 6 years. The mean age was similar between the video and control groups (5.6 ± 1.2 versus 5.8 ± 1.1 years; t = 0.72, df = 64, p = 0.47, 95% CI: -0.4 to 0.9; Cohen’s d = 0.16, negligible effect). Gender distribution was balanced (χ² = 0.18, df = 1, p = 0.67). 

**Table 2:** Age distribution of the participants Values are counts. Mean age comparison: t = 0.72, df = 64, p = 0.47, 95% CI (-0.4 to 0.9), Cohen’s d = 0.16 (negligible effect) CI: confidence interval

Age group (years)	Number of participants
4-6	41
7-8	17
9-10	8
Total	66

**Figure 3:**
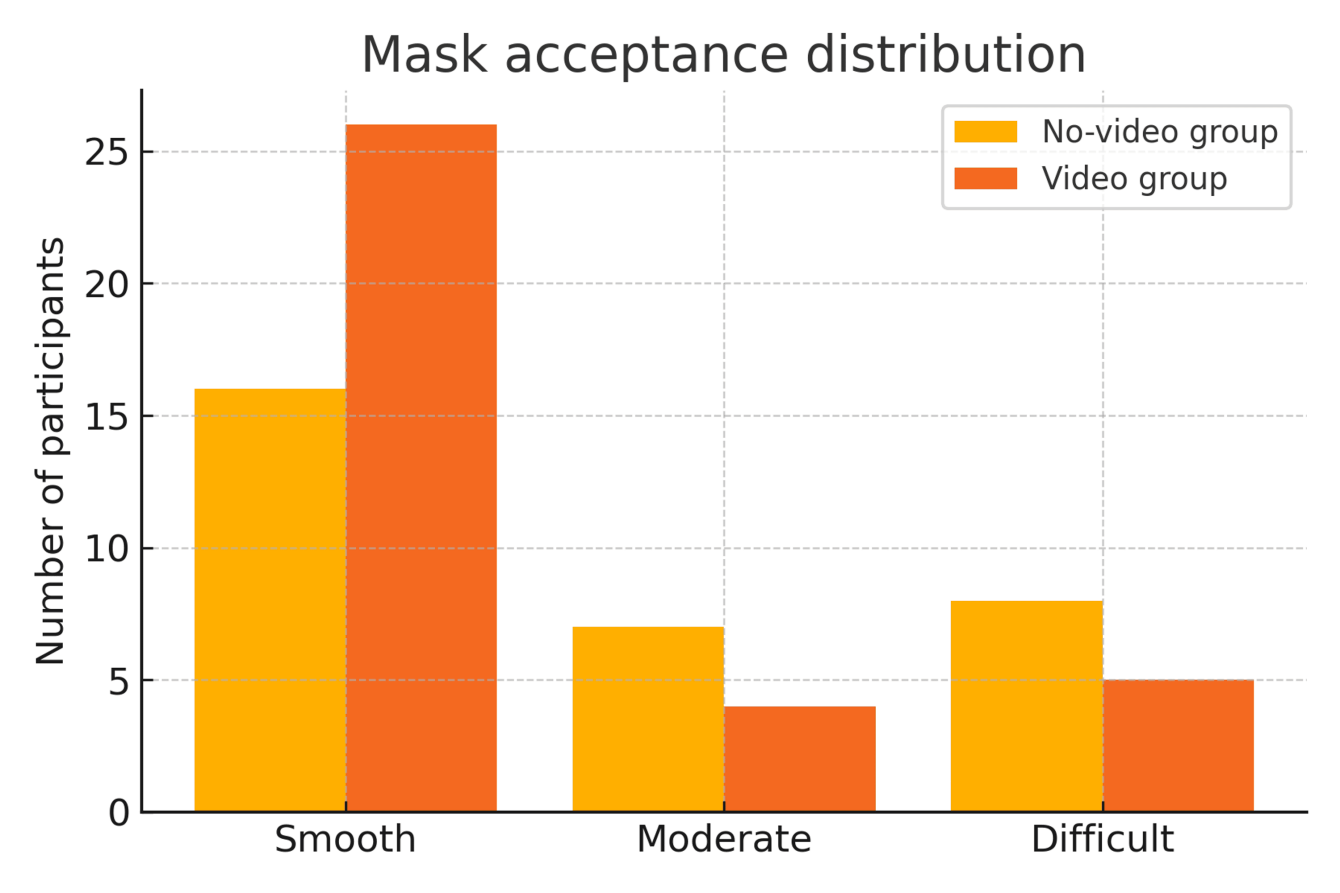
Mask acceptance distribution among the participant children in the video and no-video groups

Mask acceptance was assessed using the Child Induction Behavioural Assessment Tool (CIBAT), a validated behavioural scale developed by Winterberg et al. (2018) [14]. This tool categorises children’s behavioural responses during inhalational induction as smooth, moderate or difficult, based on cooperation, crying and resistance (Table 1). Overall, 42 of 66 children (63.6%) demonstrated smooth induction behaviour, defined by calm and cooperative acceptance of the facemask according to the Child Induction Behavioural Assessment Tool (CIBAT). Smooth induction was observed in 74.3% (26/35) of children in the video group and 51.6% (16/31) in the no-video group. A comparison between the video and no-video groups is presented in Table 3, and the distribution is illustrated in Figure 3. No statistically significant difference in mask acceptance was observed between groups (χ² = 5.12, df = 3, p = 0.16; Cramer’s V = 0.18, small effect size).

**Table 3:** Mask acceptance by group χ² = 5.12, df = 3, p = 0.16, Cramer’s V = 0.18 (small effect)

Category	Video group (n = 35)	No-video group (n = 31)	p-value
1	10	6	
2	9	6	
3	11	14	
4	5	5	
Total	35	31	0.16

The duration from application of the face mask to induction end-points is shown in Table 4. The mean time to loss of eyelash reflex (T₁) was significantly longer in the video group (237.1 ± 34.4 seconds) compared with the no-video group (217.3 ± 36.9 seconds; p = 0.027). The mean time from loss of eyelash reflex to successful intravenous cannulation (T₂) was comparable between groups (88.3 ± 13.2 seconds versus 87.0 ± 15.0 seconds; p = 0.70), indicating no difference in readiness for IV access once adequate depth of anaesthesia was achieved.

**Table 4:** Induction times Data is expressed as mean ± SD. T₁: t = 2.28, df = 64, p = 0.027, 95% CI (2.4 to 37.2), Cohen’s d = 0.56 (moderate effect) T₂: t = 0.38, df = 64, p = 0.70, 95% CI (-5.5 to 8.1), Cohen’s d = 0.09 (negligible effect) T₁: time to loss of eyelash reflex, T₂: time from loss of eyelash reflex to successful IV cannulation, SD: standard deviation, CI: confidence interval, IV: intravenous

Parameter	Video group (n = 35)	No-video group (n = 31)	p-value
Time to loss of eyelash reflex (T₁, seconds)	237.1 ± 34.4	217.3 ± 36.9	0.027
Time taken from loss of eyelash reflex to successful IV cannulation (T₂, seconds)	88.3 ± 13.2	87 ± 15.0	0.70

Category-wise analysis of induction times is presented in Table 5. Children with smooth inductions had the longest T₁ (245.7 ± 34.5 seconds) compared with moderate (203.3 ± 4.6 seconds) and difficult inductions (190.5 ± 5.1 seconds). This difference was statistically significant (p = 0.04), indicating that calmer and more cooperative children required slightly longer times to reach loss of eyelash reflex, reflecting a smoother inhalational induction process.

**Table 5:** Category-wise duration of T1 and T2 Category-wise duration of time taken for loss of eyelash reflex (T₁) and from loss of eyelash reflex to successful IV cannulation (T₂) IV: intravenous, SD: standard deviation

Category (T₁)	T₁	Mean duration (seconds)	SD	Standard error
Smooth	42	245.71	34.453	5.316
Moderate	11	203.27	4.606	1.389
Difficult	13	190.46	5.142	1.426
Total	66	227.76	36.673	4.514
Category (T₂)	T₂	Mean duration (seconds)	SD	Standard error
Smooth	42	87.17	14.201	2.191
Moderate	11	94.45	13.721	4.137
Difficult	13	83.77	13.676	3.793
Total	66	87.71	14.202	1.748

The mean T₂ (time from loss of eyelash reflex to successful IV cannulation) did not differ significantly among groups (87.2 ± 14.2 seconds for smooth, 94.5 ± 13.7 seconds for moderate and 83.8 ± 13.7 seconds for difficult inductions), demonstrating comparable IV access readiness once adequate anaesthetic depth was achieved.

**Figure 4:**
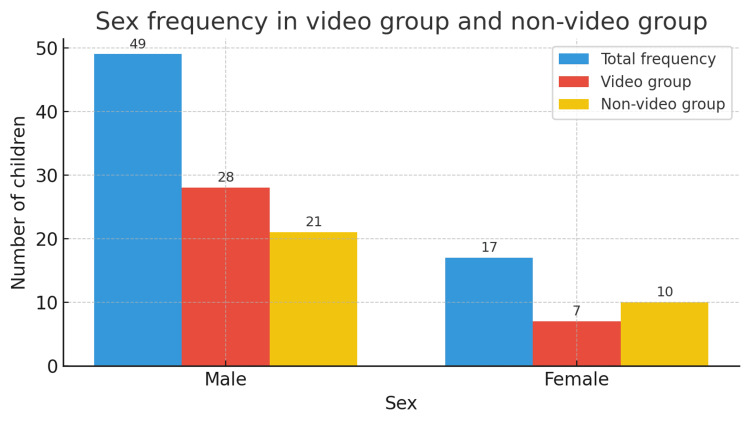
Sex distribution of the participants in the video and no-video groups

The sex distribution of the participants is shown in Figure 4.

## Discussion

This randomised controlled trial investigated whether video-assisted mask preconditioning with parental and child simulation could improve induction behaviour during paediatric inhalational anaesthesia. Although no statistically significant difference in mask acceptance was observed between groups (Table 3, Figure 3), a higher proportion of smooth inductions occurred in the video group. Clinically, smoother inductions are important, as they reduce perioperative distress and may improve cooperation during subsequent anaesthetics [7,9,13].

The mean time to loss of eyelash reflex (T₁) was significantly longer in the video group compared with controls (Table 4). Although this may initially appear disadvantageous, the longer time to loss of eyelash reflex (T₁) observed in the video group may reflect calmer, more regular breathing behaviour typically associated with cooperative induction. However, this interpretation should be viewed cautiously, as no concurrent physiological data, such as respiratory rate, tidal volume or end-tidal sevoflurane concentration, were recorded to confirm this mechanism. It is also possible that variations in mask seal, breathing depth or transient resistance could have contributed to the observed difference.

Category-wise analysis supports this behavioural interpretation: smoother inductions were associated with longer T₁, whereas difficult inductions corresponded to shorter T₁ values (Table 5). Future studies incorporating objective respiratory monitoring would help validate the relationship between behavioural calmness and induction duration. This interpretation is consistent with the physiological principles described by Lejus et al. [12], who observed that the tidal volume technique produces a slower onset compared with the single-breath vital capacity method, and with Aydin et al. [8], who demonstrated that crying and distress accelerate anaesthetic uptake through increased ventilation. Therefore, the differences in T₁ and T₂ observed in our study likely reflect variations in child behaviour and respiratory pattern, rather than methodological bias.

Our results add to existing evidence on non-pharmacological strategies for reducing perioperative anxiety. Previous studies have examined distraction with video games [16], use of flavoured masks [5] and parental presence [9], with varying outcomes.Walker et al. reported that parent-led mask exposure improved induction compliance [13], while Berghmans et al. found that viewing an audiovisual aid immediately before induction reduced parental anxiety but had limited impact on children’s mask acceptance [7]. The present trial, by combining video demonstration with parental and child simulation, aligns with this body of literature and highlights the potential for multimodal strategies rather than reliance on a single approach.

Strengths of this study include its randomised controlled design, use of a validated behavioural tool (Child Induction Behavioural Assessment Tool (CIBAT) [14]) and pragmatic incorporation of parental participation, which increases external validity.

Limitations

Several limitations must be acknowledged. First, the study was underpowered due to reduced recruitment during the COVID-19 pandemic, which limited the ability to detect statistically significant differences. Second, although the design was single-blind, only the assessor was blinded; children and parents were aware of group allocation, introducing performance bias. Third, inductions were carried out in a standard operating theatre rather than a child-friendly environment, which may have increased baseline anxiety. Fourth, the intervention was delivered only once on the evening before surgery, without reinforcement on the day of the procedure. Fifth, nitrous oxide and flavoured anaesthesia masks were not available, both of which may influence mask acceptance [5]. Sixth, baseline anxiety and temperament were not formally assessed, despite being known predictors of induction behaviour [11]. Finally, behavioural assessment was conducted at a single time point, limiting evaluation of dynamic changes during induction.

The reduced recruitment compared with the initial sample size target represents an important limitation of this study and may have limited the statistical power to detect significant differences in the primary outcome. Consequently, the absence of a statistically significant difference in overall mask acceptance should be interpreted with caution. Nevertheless, the consistent trend toward smoother inductions and improved behavioural scores in the video group suggests a potential positive effect that warrants confirmation through larger, adequately powered multicentre studies.

Summary

In summary, video-assisted mask preconditioning with parental and child simulation did not significantly improve overall mask acceptance but was associated with smoother inductions and longer times to loss of eyelash reflex. These findings are preliminary and should be interpreted cautiously, given the study’s limitations, including reduced sample size, single-centre design and the non-child-friendly operating theatre environment. Larger, adequately powered multicentre studies using multimodal strategies and conducted in child-focused settings are needed to clarify whether this approach offers meaningful benefits in routine paediatric anaesthetic practice.

## Conclusions

Video-assisted mask preconditioning with parental and child simulation was associated with a higher proportion of smooth inductions and longer time to loss of eyelash reflex; however, it did not produce a statistically significant improvement in overall facemask acceptance. These preliminary findings should be interpreted with caution, given the reduced sample size, single-centre design and non-child-friendly setting. Larger, adequately powered multicentre studies using multimodal strategies are warranted to confirm these results before any consideration of routine clinical use.
